# The Relationship between BMI and Glycated Albumin to Glycated Hemoglobin (GA/A1c) Ratio According to Glucose Tolerance Status

**DOI:** 10.1371/journal.pone.0089478

**Published:** 2014-02-28

**Authors:** Ji Hye Huh, Kwang Joon Kim, Byung-Wan Lee, Dong Wook Kim, Eun Seok Kang, Bong Soo Cha, Hyun Chul Lee

**Affiliations:** 1 Division of Endocrinology and Metabolism, Department of Internal Medicine, Yonsei University College of Medicine, Seoul, Korea; 2 Severance Executive Healthcare Clinic, Severance Hospital, Seoul, Korea; 3 Division of Medical Statistics, Yonsei University College of Medicine, Seoul, Korea; University of Catanzaro Magna Graecia, Italy

## Abstract

Glycated albumin to glycated hemoglobin (GA/A1c) ratio is known to be inversely related with body mass index (BMI) and insulin secretory capacity. However, the reasons for this association remain unknown. We aimed to investigate whether BMI directly or indirectly influences GA/A1c by exerting effects on insulin secretion or resistance and to confirm whether these associations differ according to glucose tolerance status. We analyzed a total of 807 subjects [242 drug-naïve type 2 diabetes (T2D), 378 prediabetes, and 187 normal glucose tolerance (NGT)]. To assess the direct and indirect effects of BMI on GA/A1c ratio, structural equation modeling (SEM) was performed. GA/A1c ratio was set as a dependent variable, BMI was used as the independent variable, and homeostasis model assessment-pancreatic beta-cell function (HOMA-β), homeostasis model assessment-insulin resistance (HOMA-IR), glucose level were used as mediator variables. The estimates of a direct effect of BMI on GA/A1c to be the strongest in NGT and weakest in T2D (−0.375 in NGT, −0.244 in prediabetes, and −0.189 in T2D). Conversely, the indirect effect of BMI on GA/A1c exerted through HOMA-β and HOMA-IR was not statistically significant in NGT group, but significant in prediabetes and T2D groups (0.089 in prediabetes, −0.003 in T2D). It was found that HOMA-β or HOMA-IR indirectly influences GA/A1c in T2D and prediabetes group through affecting fasting and postprandial glucose level. The relationship between GA/A1c and BMI is due to the direct effect of BMI on GA/A1c in NGT group, while in T2D and prediabetes groups, this association is mostly a result of BMI influencing blood glucose through insulin resistance or secretion.

## Introduction

Until now, the gold standard parameter for monitoring glycemic excursion has been glycated hemoglobin (A1c). However, A1c does not provide accurate information following earlier changes in glycemic control after drug intervention or in various conditions affecting the lifespan of red blood cells [1]–[3]. Although glycated albumin (GA), a useful glycemic index for intermediate periods over 2–4 weeks, may be viewed as an adjunct to A1c, it is gaining popularities during the transition between medications for intensive treatment or for diabetes management at a monthly level [4]–[6]. In addition, serum GA has been shown to be a superior indicator for plasma glucose variability to A1c [7].

Recently, not only GA but also the ratio of GA to A1c (GA/A1c) is expected to be a new glucose control marker [8]. However, notwithstanding the pathologic condition affecting albumin metabolism such as thyroid dysfunction, nephrotic syndrome, or liver cirrhosis [4], the physiologic variables such as age or body mass index (BMI) [9] make the GA/A1c ratio a little unpredictable in clinical practice. Among them, several studies have suggested a negative correlation between BMI and serum GA in non-diabetic children, as well as in adult diabetic patients [9]–[11]. However, conflicting result was observed in Kyushu and Okinawa Population Study, which reported no significant association between GA and BMI in type 2 diabetic subjects [12]. In spite of the discordant results on the association between GA/A1c ratio and BMI in especially diabetic patients, there has been no study in the literature to date focusing on the relationship between GA/A1c ratio and BMI according to glucose tolerance status. Moreover, we have demonstrated that insulin secretory functions, such as homeostasis model assessment-pancreatic beta-cell function (HOMA-β) and insulinogenic index, but not insulin resistance, are negatively associated with GA/A1c ratio in patients with type 2 diabetes (T2D) [13]. Increase in BMI generally leads to not only insulin resistance but also compensatory elevated insulin secretion, and insulin secretion is inversely associated with GA/A1c; therefore, a decrease in GA/A1c is expected following elevated BMI. In other words, it could be hypothesized that the influence of BMI on GA/A1c level might be mediated through elevated insulin secretion. However, there are few studies reporting the relationship between GA/A1c ratio and BMI in association with insulin secretory function, insulin resistance, and serum glucose level. Therefore, this study aimed to observe the association between BMI, insulin secretion, resistance, blood glucose, and GA/A1c according to glucose tolerance; furthermore, it employed structural equation modeling (SEM), which can differentiate direct and indirect effects, to identify whether the characteristics of aforementioned associations are different among subjects with normal glucose tolerance, prediabetes, and T2D.

## Materials and Methods

### Ethics Statement

The study was carried out according to the Declaration of Helsinki and the International Conference on Harmonization Good Clinical Practice Principles. The protocol was approved by the independent institutional review board at Yonsei University College of Medicine. All enrolled subjects provided written informed consent.

### Study population and design

In this clinical, cross-sectional study, we analyzed patients who satisfied certain criteria based on their medical records. We included patients enrolled in the diabetes registry of Severance Diabetes Center between June 2008 and February 2012; only first-time visitors to the center and subjects who had been tested for GA, HbA1c, plasma glucose and C-peptide were included. Exclusion criteria included a history of use of hypoglycemic or lipid-lowering agents, severe liver or kidney disease (chronic kidney disease ≥ stage 3), active thyroid disorders, pregnancy, steroid therapy, heavy alcohol usage, Type 1 diabetic patients (C-peptide <0.5 ng/mL) and malignant disease.

To investigate the relationship between BMI and GA/A1c ratio stratified by degree of insulin secretory function and insulin resistance, patients were classified into 3 groups based on the American Diabetes Association 2011 guidelines: T2D (A1c≥6.5%), increased risk for diabetes (A1c = 5.7–6.4%, described as prediabetes hereon), and normal glucose tolerance (NGT) (A1c≤5.6%) [3], [14]. Anthropometric measurements were taken with patients wearing light clothing and no shoes. Waist circumference was measured with the tape measure placed horizontally at the level of the umbilicus while the participant gently exhaled. BMI was calculated as weight in kilograms divided by the square of height in meters. The study protocol was approved by the Ethics Committee of Yonsei University College of Medicine.

### Laboratory measurements

Blood samples were collected at 0 and 90 mins (postprandial) for glucose, insulin and C-peptide analyses. Plasma glucose levels were measured using the glucose oxidase method (Hitachi 747 automatic analyzer, Hitachi Instruments Service, Tokyo, Japan). Serum GA levels were measured using the enzymatic method and a Hitachi 7699 P module autoanalyzer (Hitachi Instruments Service). A1c levels were measured by high-performance liquid chromatography using a Variant II Turbo (Bio-Rad Laboratories, Hercules, CA, USA). Serum insulin and C-peptide levels were measured in duplicate by immunoradiometric assay (Beckman Coulter, Fullerton, CA, USA).

Basal β-cell function and insulin resistance were assessed by HOMA-β and insulin resistance was assessed by homeostasis model assessment-insulin resistance (HOMA-IR).
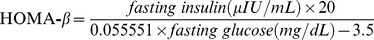


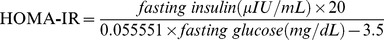



### Statistical analyses

All continuous variables are shown as mean ± standard deviation. Analysis of variance (ANOVA) followed by post hoc analysis using Bonferroni correction was used to compare variables across multiple groups. The relationships between clinical and laboratory variables were evaluated using univariate Pearson's correlation analysis. To correct for skewed distributions, HOMA-β, HOMA-IR were logarithmically transformed. Then SEM analyses were performed to assess the direct and indirect effects of between variables. Values of variables used in SEM were standardized due to the wide ranges encountered for these variables. The standardization method for a dataset of size *k* with mean value *μ* and variance *σ^2^* is as follows:
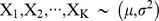


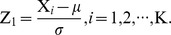




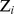
 is a standardized value based on the mean and standard deviation.

**Figure 1:**
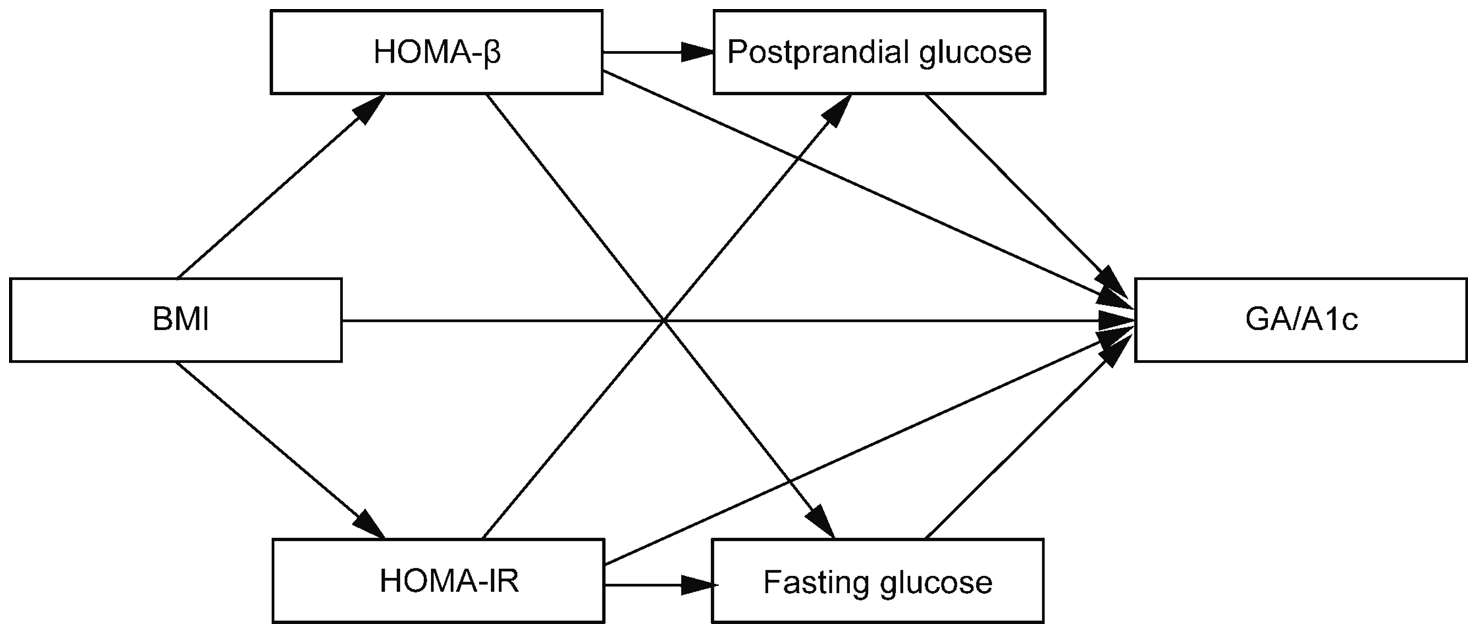
Structural equation models for the GA/A1c ratio.

To assess the direct effect of BMI on GA/A1c ratio independent of the indirect effect of BMI on GA/A1c ratio mediated by other variables, a statistical analysis was performed using SEM and path diagram analysis by IBM® SPSS® Amos (Figure S1) [15]. Briefly, GA/A1c ratio was set as a dependent variable, and BMI was set as an independent variable. HOMA-β, HOMA-IR, fasting glucose and postprandial glucose were used as mediator variables which are associated with each other and with GA/A1c ratio, respectively. The purpose of this analysis was to observe whether the independent variables—in this case, BMI—have direct or indirect effects (through mediator variables) on GA/A1c ratio, the dependent variable. In other words, although one independent variable may seem to directly influence GA/A1c ratio, a path diagram analysis may reveal that this relationship is in fact due to another independent variable that acts as a mediator between the first independent variable and GA/A1c ratio. For this study, we proposed the following path diagrams (Figure 1) which was examined for each group. *P* values less than 0.05 were considered statistically significant. All data were analyzed using PASW Statistics version 18 (SPSS Inc, Chicago, IL, USA).

## Results

### Baseline Characteristics of Patients

A total of 807 patients (242 with T2D, 378 with prediabetes, and 187 with NGT) satisfied the inclusion criteria of this study. Table 1 summarizes the demographic and clinical characteristics of the patients. The mean age of all patients was 55.26 years, and the mean GA/A1c ratio was 2.20±0.41. BMI, waist-hip ratio, and body weight were significantly higher in the T2D group, followed by the prediabetes and the NGT groups. GA and GA/A1c ratio were significantly higher in the T2D group than other two groups. HOMA-β (%) was significantly higher (*P*<0.001) in the prediabetes group (80.55±54.17), followed by the NGT (76.96±54.10) and the T2D (53.86±55.61) groups. HOMA-IR was significantly higher (*P*<0.001) in the T2D group (3.61±3.04), followed by the prediabetes (1.93±1.40) and the NGT (1.27±0.84) groups.

**Table 1:** Baseline characteristics.

	Total N = 807	NGT n = 187	Prediabetes n = 378	Diabetes n = 242	P value
Sex: M/F (male %)	432/375 (53.5%)	93/94 (49.7%)	198/180 (52.4%)	141/101 (58.3%)	0.177
Age (year)	55.26±11.51	49.02±11.92† ‡	58.19±9.84† #	55.53±11.76‡ #	<0.001
Height (cm)	165.04±8.52	166.05±8.58	164.80±8.15	164.62±9.01	0.171
Weight (Kg)	67.19±12.48	63.85±11.92† ‡	67.09±12.31† #	69.93±12.55‡ #	<0.001
BMI (kg/m2)	24.58±3.45	23.05±2.96† ‡	24.60±3.40† #	25.75±3.41‡ #	<0.001
WHR	0.89±0.06	0.86±0.08† ‡	0.89±0.05† #	0.91±0.05‡ #	<0.001
SBP (mmHg)	126.14±16.68	119.16±13.91† ‡	125.63±15.92† #	132.29±17.53‡ #	<0.001
DBP (mmHg)	78.41±11.15	76.44±10.23‡	78.21±11.06	80.21±11.72‡	0.002
HbA1c (%)	6.55±1.52	5.47±0.16† ‡	5.98±0.22† #	8.27±1.81‡ #	<0.001
GA (%)	14.76±6.28	11.27±1.48† ‡	12.34±1.77† #	21.10±8.07‡ #	<0.001
GA/A1c	2.20±0.41	2.06±0.28‡	2.06±0.27#	2.52±0.52‡ #	<0.001
Glucose at 0 min (mg/dL)	111.57±36.93	88.98±8.14† ‡	99.30±11.27† #	147.34±49.25‡ #	<0.001
Glucose at 90 min (mg/dL)	164.32±78.34	112.79±33.61† ‡	135.21±44.55† #	241.04±81.96‡ #	<0.001
C-peptide at 0 min (µg/L)	2.26±1.12	1.83±0.92† ‡	2.24±1.08† #	2.56±1.19‡ #	<0.001
C-peptide at 90 min (µg/L)	7.15±3.66	6.63±4.08†	7.75±3.77† #	6.52±3.12#	<0.001
Insulin at 0 min (µIU/mL)	7.98±5.91	5.55±3.60† ‡	7.71±5.13† #	9.83±7.39‡ #	<0.001
Insulin at 90 min (µIU/mL)	50.63±46.97	35.28±40.39† ‡	52.56±50.24†	55.41±43.47‡	<0.001
HOMA-β (%)	71.49±55.85	76.96±54.10‡	80.55±54.17#	53.86±55.61‡ #	<0.001
HOMA-IR	2.34±2.20	1.27±0.84† ‡	1.93±1.40† #	3.61±3.04‡ #	<0.001
Cholesterol (mg/dL)	188.83±38.92	186.17±35.10	188.90±38.84	190.74±41.74	0.488
LDL (mg/dL)	110.23±35.19	109.82±30.74	112.68±34.94	106.69±38.47	0.117
HDL (mg/dL)	51.26±19.27	52.18±13.67	50.82±17.39	51.23±25.00	0.735
Fasting TG (mg/dL)	131.32±98.59	105.75±69.75‡	117.80±57.77#	172.37±145.27‡ #	<0.001
Postprandial TG (mg/dL)	156.86±117.63	104.43±84.12‡	124.45±63.15#	206.91±147.97‡ #	<0.001
Hb (g/dL)	14.21±1.49	14.13±1.42	14.10±1.49#	14.47±1.54#	0.01
Protein (g/dL)	6.95±0.42	6.80±0.37† ‡	6.92±0.41† #	7.11±0.43‡ #	<0.001
Albumin (g/dL)	4.37±0.33	4.30±0.32‡	4.33±0.29#	4.49±0.35‡ #	<0.001
Creatinine (mg/dL)	0.84±0.31	0.77±0.17† ‡	0.86±0.40†	0.87±0.22‡	0.003

Data presented as n (%) or mean ± standard deviation.

† : The difference between NGT and Prediabetes : p<0.05 after Bonferroni correction.

‡ : The difference between NGT and Diabetes : p<0.05 after Bonferroni correction.

# : The difference between Prediabetes and Diabetes : p<0.05 after Bonferroni correction.

Abbreviations: NGT, normal glucose tolerance; BMI, body mass index; WHR, waist-to-hip ratio; SBP, systolic blood pressure; DBP, diastolic blood pressure; HbA1c, glycated hemoglobin; GA, glycated albumin; GA/A1c, ratio of glycated albumin to glycated hemoglobin; HOMA-β, homeostasis model assessment- pancreatic beta-cell function; HOMA-IR, homeostatsis model assessment-insulin resistance; LDL, low-density lipoprotein; HDL, high-density lipoprotein; TG, triglyceride.

### Correlation of GA/A1c Ratio and BMI in Diabetic, Prediabetic, and NGT Patients

To assess the correlation between GA/A1c ratio and BMI according to glucose tolerance status, we performed Pearson correlation analysis (Table 2). The negative association between GA/A1c ratio and BMI was the most prominent in the NGT group. (NGT: R = −0.383, *P*<0.001; prediabetes: R = −0.221, *P*<0.01; T2D: R = −0.181, *P*<0.001). There was a significant positive correlation between fasting, postprandial glucose and GA/A1c ratio in the prediabetes and the T2D groups. Moreover, a negative relationship between GA/A1c ratio and HOMA-β was significant in the T2D group and the prediabetes groups but not in the NGT group (prediabetes: R = −0.342, *P*<0.001; and T2D: R = −0.322, *P*<0.001). The relationship between insulin resistance and GA/A1c ratio was statistically significant only in the prediabetes groups (R = −0.138, *P* = 0.007).

**Table 2:** Correlations between GA/A1c ratio and other variables.

	GA/A1c		
Variables	NGT (*n* = 187)	Prediabetes (*n* = 378)	Diabetes (*n* = 242)
Age	NS	0.151(0.003)	NS
WHR	NS	−0.178(0.001)	NS
BMI	−0.383(<0.001)	−0.221(<0.001)	−0.181(0.005)
A1c	−0.183(0.015)	0.188(<0.001)	0.423(<0.001)
Glycated albumin	0.971 (<0.001)	0.963 (<0.001)	0.807(<0.001)
Glucose at 0 min	NS	0.293(<0.001)	0.459(<0.001)
Glucose at 90 min	NS	0.258(<0.001)	0.463(<0.001)
LN HOMA-β	NS	−0.342 (<0.001)	−0.332(<0.001)
LN HOMA-IR	NS	−0.138 (0.007)	NS

Values are Pearson correlation coefficients between variables and GA/A1c ratio.

Abbreviations: NGT, normal glucose tolerance; BMI, body mass index; WHR, waist-to-hip ratio; A1c, hemoglobin A1c; GA/A1c, ratio of glycated albumin to glycated hemoglobin; LN HOMA-β, log transformed homeostasis model assessment-pancreatic beta-cell function; LN HOMA-IR, log transformed homeostatis model assessment-insulin resistance; NS, not significant.

### Decomposition of Direct and Indirect Effect of BMI on GA/A1c Ratio

**Figure 2:**
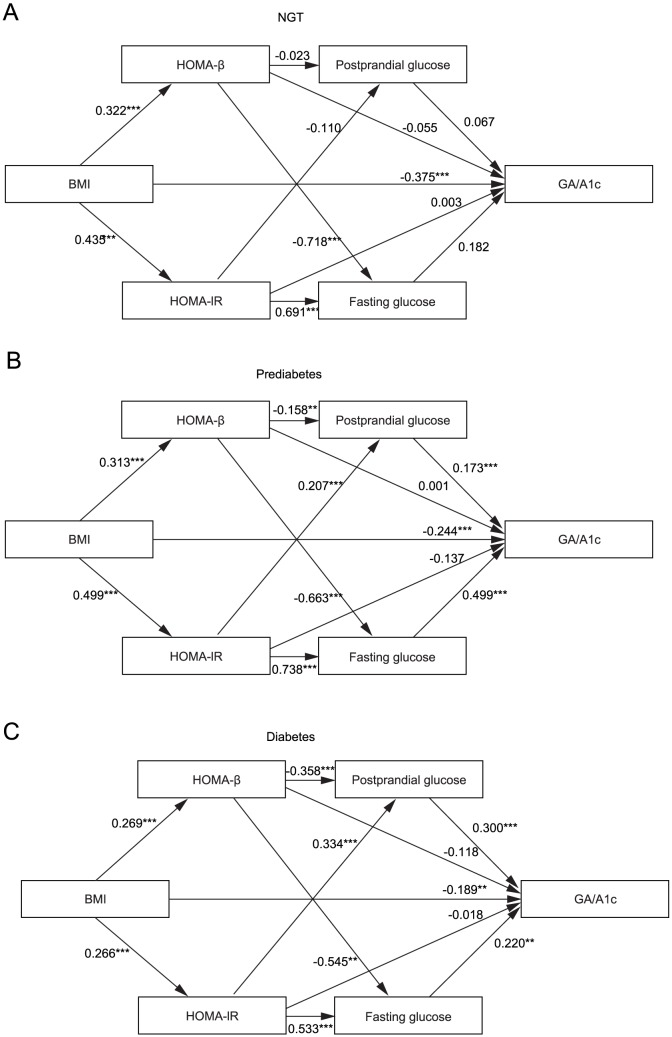
Structural equation models for the GA/A1c ratio in NGT (A), prediabetes (B) and diabetes group (C). *P<0.05; **P<0.01;*** *P*<0.001.

SEM was employed to separately analyze the direct effects of BMI on GA/A1c ratio (Figure 1). Figure 2 depicts the SEM for GA/A1c ratio in each glucose tolerance group. Using this model, the estimates of a direct effect of BMI on GA/A1c were −0.375 (*P*<0.001) in the NGT group, −0.244 (*P*<0.001) in the prediabetes group, and −0.189 (*P* = 0.002) in the T2D group. In the NGT group, there was no significant indirect effect of BMI on GA/A1c ratio. In contrast, in prediabetes and T2D group, the indirect effects of BMI on GA/A1c ratio which is mediated by HOMA-β→fasting glucose or postprandial glucose and HOMA-IR→fasting glucose or postprandial glucose were significant. However, in the prediabetes and the T2D groups, these indirect effects of BMI on GA/A1c ratio were relatively weak (0.089 in the prediabetes group; −0.003 in the T2D group). In each group, the variables which were significantly associated with GA/A1c ratio was only BMI in the NGT group (estimate of effect: −0.375, P<0.001), while fasting glucose, postprandial glucose, and BMI in the prediabetes and T2D groups. In addition, in prediabetes and T2D group, the influence of fasting and prostprandial glucose parameters on GA/A1c was greater than that of BMI. (BMI→GA/A1c: −0.244, Postprandial glucose→ GA/A1c: 0.173, Fasting glucose→ GA/A1c: 0.499 in prediabtes group; BMI→GA/A1c: −0.189, Postprandial glucose→ GA/A1c: 0.300, Fasting glucose→ GA/A1c: 0.220 in T2D group). The degree of effect of BMI on HOMA-β was the most prominent in NGT, followed by the prediabetes and the T2D groups, in that order. However, a direct effect of HOMA-β or HOMA-IR on GA/A1c was not observed in any groups.

## Discussion

The present study represents the first of its kind to investigate the relation between BMI and GA/A1c according to glucose tolerance status. Our results indicated that inverse association between BMI and GA/A1c ratio was observed in all glucose tolerance status, which was strongest in NGT group, followed by the prediabetes and the T2D groups, in that order. Furthermore, using SEM, we found that the variables influencing GA/A1c ratio was different according to glucose tolerance status; only BMI in the NGT group, BMI, postprandial glucose and fasting glucose in the prediabetes and T2D groups. The results suggested that although it is true that BMI is inversely related to GA/A1c ratio, the ratio is under greater influence by glucose parameters than by BMI in prediabetes or T2D; therefore, this suggests that while GA/A1c cannot be an accurate index of glycemic control status in NGT, it may be a significant index in prediabetes or diabetes regardless of BMI.

Previous studies have indicated that obesity is negatively associated with GA and GA/A1c ratio. However, the underlying mechanisms of this relationship remain to be answered, and they were not fully evaluated in subjects with prediabetes or NGT. Koga et al. demonstrated that obesity and its related chronic inflammation are involved in lower serum GA levels [16]. On the other hand, other studies have suggested that the negative association of obesity with GA is due to abnormal albumin concentrations in obese subjects [17]. However, Nishimura et al indicated that obese children had higher serum albumin than non-obese children, and Koga et al. found no correlation between BMI and albumin concentrations [9], [16]. Based on these unclear answers for the mechanism of the association between BMI and GA/A1c ratio, we tried to explain this mechanism with respect to BMI, a representative parameter for obesity, and insulin secretory function. In accordance with the increase of BMI, insulin secretory function might be also increased to overcome the insulin resistance. However, the degree of increase of insulin secretory function required to overcome insulin resistance would differ according to various glucose tolerance status. Therefore, the effect of BMI on GA/A1c, which is negatively associated with insulin secretory function [18], would also differ according to glucose tolerance status. Based on these findings, we hypothesized that the magnitude of negative influence of BMI on GA/A1c ratio might be dependent on gluco-insulin homeostasis, especially on insulin secretory function compensating for insulin resistance. Therefore, we investigated the association between BMI and GA/A1c ratio according to glucose tolerance status. To address these questions, we recruited drug-naive subjects with NGT, prediabetes, and T2D. The present study represents the first of its kind to investigate the relation between BMI and GA/A1c ratio according to glucose tolerance status. The results demonstrated four main findings with respect to correlations with the GA/A1c ratio.

First, the inverse association between BMI and GA/A1c ratio was observed in all glucose tolerance status, which was strongest in NGT group, followed by the prediabetes and the T2D groups. The reason for the different degrees of effect of BMI on GA/A1c ratio by glucose tolerance status groups may be a greater influence of BMI on factors other than GA/A1c ratio in the prediabetes and the T2D groups. A recent, large population-based study [12] showed that BMI had no effect on GA levels in subject with T2D, even when including patients with high BMI. This unexpected result suggests that BMI exerts influence on other factors besides GA; consequently, the association between BMI and GA may seem to have been weakened especially in subjects with T2D. Second, using SEM analysis, we found that HOMA-β does not significantly affect GA/A1c. Similar to previous studies [14], a simple correlation analysis found a negative correlation between HOMA-β and GA/A1c ratio. However, when we analyzed SEM which excluded other factors that influences GA/A1c ratio, we observed that HOMA- β does not directly affect GA/A1c ratio. These findings explain that the negative association between HOMA-β and GA/A1c ratio shown in previous studies [13], [14] may be attributed to the indirect effect of the glucose variability of fasting and postprandial glucose mainly caused by the decline of insulin secretory function [19] Third, the variables influencing GA/A1c ratio were different according to glucose tolerance status groups. In the NGT group, only BMI significantly influenced GA/A1c ratio, whereas in the prediabetes and the T2D groups, fasting glucose and postprandial glucose also influenced GA/A1c ratio. The absence of influence of glucose level in the NGT group may be explained by the following. The pathophysiology of T2D is characterized by insulin resistance and impaired compensatory insulin secretion. Therefore, in T2D, insulin secretion is unable to quickly compensate for insulin resistance, leading to increased postprandial sugar levels and greater elevation of GA (known to be an index reflecting postprandial glycemic status) compared to A1c. However, in patients with NGT with intact insulin secretory function, excessive postprandial elevation of blood glucose is not observed, and a disproportionate increase of GA compared to A1c does not occur, resulting in loss of association between GA/A1c ratio and insulin secretory function. Furthermore, we have confirmed that only BMI held a significant influence over GA/A1c ratio in NGT while in prediabetes and T2D, both glucose parameters and BMI significantly inflenced GA/A1c ratio, with the former providing a greater influence. These findings may be the basis of explaining the clearer effect of BMI on GA/A1c ratio in NGT group. Fourth, the effect of HOMA-β on fasting glucose was the most prominent in the NGT group, followed by the prediabetes group and the T2D groups. Also, the effect of HOMA-β on postprandial glucose was the most prominent in the T2D group, followed by the prediabetes and the NGT groups. This result is consistent with the previous study because early-phase insulin secretion and glucose-stimulated insulin secretion are decreased in the NGT and the prediabetes groups, which mainly contribute to the increase of fasting glucose [20]. On the other hand, because the overall insulin secretory function declines in T2D group, the insulin secretory function might influence the postprandial glucose as well as the fasting glucose compared with other groups. These findings provide important clues to the greater understanding of the concept of GA/A1c ratio with reference to glucose parameter.

A recent study has demonstrated that GA may be lower than the actual plasma glucose levels in NGT patients who have elevated body fat content; furthermore, the actual plasma glucose levels may be underestimated in obese patients when monitoring glycemic control with GA alone [21]. These data corresponded well to our result, which showed a strong association between BMI and GA/A1c ratio in NGT patients. However, in our study, the association between BMI and GA/A1c ratio was relatively weak in the prediabetes and the T2D groups. Instead, GA/A1c ratio was more strongly related with increasing glucose level. Therefore, when monitoring glycemic control with GA, it is necessary to consider the effect of BMI on GA/A1c ratio. Nonetheless, because the effect of BMI on GA/A1c ratio is relatively weaker than those of the glucose parameters in the T2D group, GA/A1c ratio well reflect the status of glucose control even in high BMI.

Our study has some limitations. First, we could not measure body composition, such as fat mass, or inflammatory cytokines (e.g. CRP), which may explain the mechanism of the negative association between BMI and GA/A1c ratio. Second, the cross-sectional study design precluded observations of future variations. Third, we could not adjust for known independent influencing factors of GA, such as triglyceride, smoking status, and age. Despite all these weaknesses, however, this study has found that the direct effect of HOMA-β alone on GA/A1c ratio is not significant in diabetic patients, contrary to the findings of previous studies which suggested an inverse association between GA/A1c ratio and HOMA-β. The previously found association between these two factors is more likely to be mediated through fasting and/or postprandial glucose. Furthermore, the factors influencing GA/A1c ratio is different according to glucose tolerance status. Insulin secretory function is preserved in the NGT, resulting in less glucose excursion; consequently, GA does not increase compared to A1c, which explains the fact that the ratio is not associated with other glucose parameters (fasting/postprandial glucose, HOMA-β, etc.) in the NGT group. Fasting glucose exerted the greatest influence on GA/A1c ratio in the prediabetes group, whereas postprandial glucose was the greatest contributing factor to GA/A1c ratio in the T2D group.

In conclusion, the inverse association between GA/A1c and BMI is the result of different mechanisms according to glucose tolerance status: in the NGT group, it is due to the direct association between BMI and GA/A1c, while in the prediabetes and T2D group, GA/A1c ratio was influenced by glucose parameters in addition to BMI, resulting in less influence of BMI on the ratio compared to NGT. These findings suggest that GA may underestimate the actual glycemic status in obese patients; however, this discrepancy tends to disappear as the subject reaches closer to T2D.

## Supporting Information

Figure S1 **Structural equation modeling (SEM).**
(TIF)
