# Supplementary figures and images for: The Relationship between BMI and Glycated Albumin to Glycated Hemoglobin (GA/A1c) Ratio According to Glucose Tolerance Status
Source: PLoS One. 2014 Feb 28;9(2):e89478. doi: 10.1371/journal.pone.0089478 (PMC3938490; doi:10.1371/journal.pone.0089478)

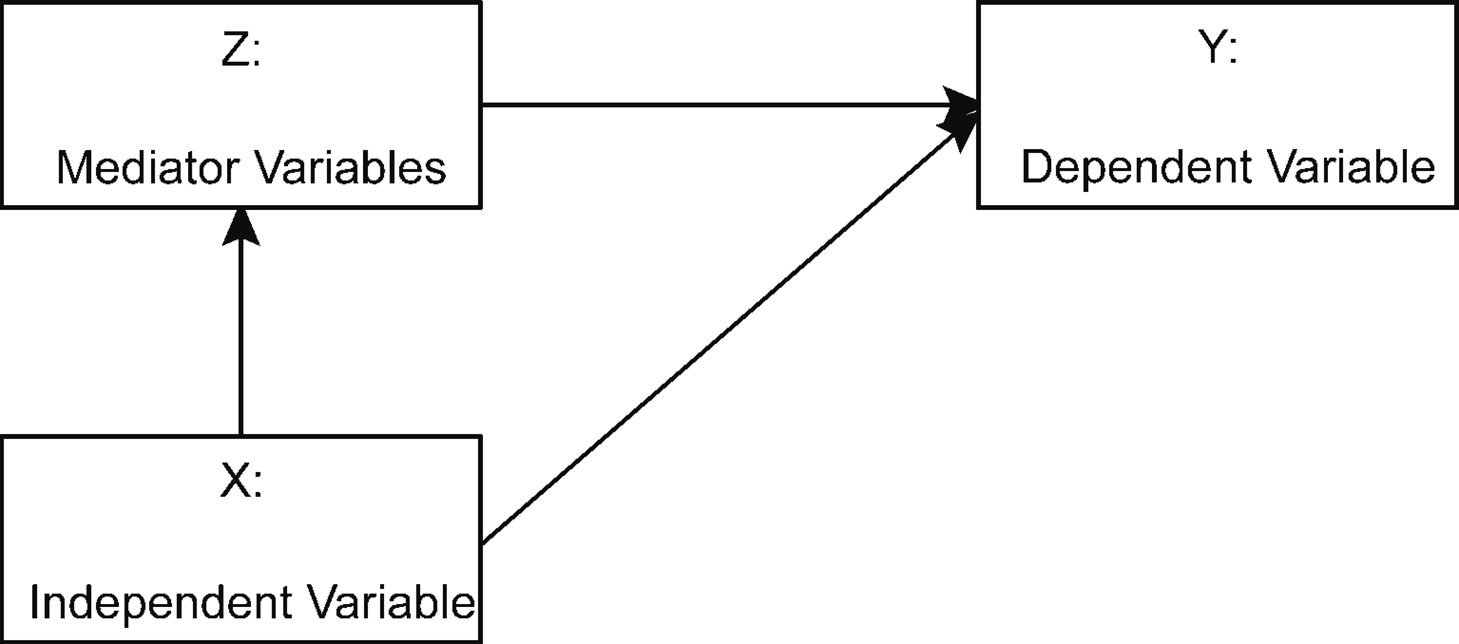

Supplement: Figure S1 — Structural equation modeling (SEM). (TIF) [file pone.0089478.s001.tif]
